# The Effectiveness of eHealth Interventions on Lifestyle Modification in Patients With Nonalcoholic Fatty Liver Disease: Systematic Review and Meta-analysis

**DOI:** 10.2196/37487

**Published:** 2023-01-23

**Authors:** Oh Young Kwon, Jin-young Choi, Yeonsoo Jang

**Affiliations:** 1 College of Nursing and Brain Korea 21 FOUR Project Yonsei University Seoul Republic of Korea; 2 College of Nursing Yonsei University Seoul Republic of Korea; 3 Severance Hospital Yonsei University Healthcare System Seoul Republic of Korea; 4 College of Nursing and Mo-Im Kim Nursing Research Institute Yonsei University Seoul Republic of Korea

**Keywords:** eHealth, lifestyle modification, non-alcoholic fatty liver disease, systematic review, meta-analysis

## Abstract

**Background:**

The prevalence of nonalcoholic fatty liver disease (NAFLD) is increasing in parallel with the epidemic of obesity and metabolic syndrome. Lifestyle modification is a crucial strategy for the treatment of NAFLD, which can lead to a reduction in liver fat with concomitant weight loss. The use of eHealth technologies is an effective approach to improve health outcomes in patients as they do not have any time and space limitations.

**Objective:**

This study aimed to evaluate published eHealth intervention studies for the improvement of lifestyle modifications among patients with NAFLD and to provide recommendations for future studies.

**Methods:**

We conducted a systematic review and meta-analysis. Five electronic databases (PubMed, Cochrane Central, CINAHL, Embase, and Web of Science) were searched for studies reporting the effect of lifestyle modification intervention using eHealth in patients with NAFLD published from inception to November 3, 2022. Study selection, data extraction, and quality assessment were performed by 3 researchers independently. The quality of included studies was assessed using the Cochrane risk of bias tool and the Risk of Bias Assessment Tool for Nonrandomized Studies.

**Results:**

In total, 2688 records were identified, and 41 full-text articles were assessed. Seven studies were included in the systematic review. The participants of all interventions were 1257 individuals with NAFLD, and the mean age ranged from 38.3 to 57.9 years. The duration of the interventions was 3-24 months, and all interventions were categorized into 3 types: internet-based computers, telephones, and mobile apps. Of these, 4 studies were randomized controlled trials and were included in the meta-analysis: 3 studies for body weight and BMI and 4 studies for alanine aminotransferase (ALT) and aspartate aminotransferase (AST). According to the meta-analysis, clear improvements in BMI (*P*=.02; 95% CI –1.01 to –0.10), AST (*P*=.02; 95% CI –1.22 to –0.13), and ALT (*P*=.01; 95% CI –1.28 to –0.15) were observed in the eHealth intervention as compared with the control groups.

**Conclusions:**

Lifestyle modification interventions using eHealth technologies are significantly effective for BMI, AST, and ALT in patients with NAFLD. Future research should conduct interventions with larger sample sizes and evaluate whether these interventions have sustained benefits, and how we can make these eHealth methods most effective on a large scale.

## Introduction

### Background

Nonalcoholic fatty liver disease (NAFLD) is a growing common cause of chronic disease. The global prevalence of NAFLD, which is associated with metabolic syndrome or other chronic diseases such as diabetes, hyperlipidemia, and hypertension, is approximately 33% [1]. NAFLD is historically defined as the presence of >5% hepatic steatosis, excluding secondary causes of hepatic fat accumulation, including excessive alcohol consumption, steatogenic medications, or hereditary disorders [2]. Clinically, NAFLD encompasses a broad spectrum of liver conditions ranging from simple steatosis or steatohepatitis to hepatic fibrosis, which may lead to cirrhosis, end-stage liver disease, or hepatocellular carcinoma [3].

NAFLD is usually asymptomatic in the majority of patients and diagnosed by imaging or histology. Since NAFLD is a progressive disease [4], patients with NAFLD have an increased risk of liver-related complications if not managed. They can develop steatohepatitis, liver fibrosis, cirrhosis, and other related complications, including variceal bleeding, ascites, hepatorenal syndrome, hepatic encephalopathy, and hepatocellular carcinoma [5,6]. In addition, if patients experience these complications, it might result in an increased mortality. However, currently, there is no approved pharmacological agent for treating NAFLD, and therefore, its sustainable management is very important.

The treatment of NAFLD has focused on lifestyle modifications related to dietary habits and physical activity leading to body weight loss [2,7]. The current guidelines recommend achieving 7%-10% weight loss as a goal for improving NAFLD conditions [2,8].

Previous studies have conducted lifestyle interventions of various durations and strategies, and several studies have reported positive effects [9-11]. However, despite these considerable efforts, changing one’s lifestyle behavior is not easy, and the majority of patients still have not reached the treatment goal, with some difficulties related to sustainability of compliance, proper social support, or constraints on time and place [12-14]. Similar to the COVID-19 pandemic, when on-site medical treatment with clinical health professionals is restricted, a more effective strategy is needed to improve patients’ health outcomes.

eHealth is defined as health services and information delivered or enhanced through the internet and related technologies [15]. As an innovative health care delivery alternative to meet the increasing demand for long-term care of chronic conditions and health care costs [16], eHealth has been presented as a progressive strategy in various fields. Meta-analyses indicate the positive effects of eHealth services on patients’ health outcomes [17,18]. This is encouraging evidence that eHealth is a beneficial approach to facilitate the modification of unhealthy behavior in patients with NAFLD. However, many eHealth interventions have reported some limitations among patients with other chronic diseases [19], and only a few studies have reported the effectiveness of lifestyle interventions for NAFLD management. Therefore, health professionals may help identify the effects of eHealth interventions and make decisions regarding intervention development and application [20].

### Objective

This systematic review aimed to evaluate the effectiveness of lifestyle modification interventions, using eHealth, in experimental trials involving adults with NAFLD. Additionally, the review compares these eHealth interventions with usual care in terms of key clinical outcome variables.

## Methods

### Protocol and Registration

This systematic review and meta-analysis was conducted using the Cochrane Handbook for Systematic Reviews of Interventions and was reported following the PRISMA (Preferred Reporting Items for Systematic Reviews and Meta-Analysis) statement guidelines [21]. This review was registered in the International Prospective Register of Systematic Reviews (registration number: CRD42021261553). All quantitative studies reporting the use of eHealth interventions were included. Studies were excluded if they did not report outcome measures or were reviews, commentaries, or qualitative studies.

### Data Sources and Search Strategy

We conducted a systematic literature search of the PubMed, Cochrane Central, Embase, CINAHL, and Web of Science databases on November 3, 2022. The search strategy included the following keywords as Medical Subject Headings (MeSH) or Embase Subject Headings terms: (“non-alcoholic fatty liver”) AND (“lifestyle modification” OR “diet” OR “exercise” OR “weight loss” OR “body mass index”) AND (“eHealth”). For example, the search strategy in PubMed is presented in Textbox 1. Multimedia Appendix 1 provides the search strategy in all databases.

**An example of the search strategy in PubMed.**

**Nonalcoholic fatty liver**

- “non-alcoholic fatty liver” [MeSH Terms] OR “non-alcoholic fatty liver” OR “nonalcoholic fatty liver” OR “non-alcoholic fatty liver” OR “NAFLD” OR “nonalcoholic steatohepatitis” OR “NASH” AND “fatty liver” [MeSH Terms] OR “fatty liver” OR “hepatosteatosis” OR “hepatic steatosis.”

**Diet, exercise, weight loss, BMI, and lifestyle modification**

- “diet” [MeSH Terms] OR “diet” OR “nutrition” [MeSH Terms] OR “nutrition” OR “food intake” [MeSH Terms] OR “food intake” OR “exercise” [MeSH Terms] OR “exercise” OR “physical activity” OR “weight loss” [MeSH Terms] OR “weight loss” OR “body weight” [MeSH Terms] OR “body weight” OR “body weight change” [MeSH Terms] OR “body weight change” OR “lifestyle modification” OR “lifestyle change” OR “body mass index” [MeSH Terms] OR “body mass index” OR “BMI.”

**eHealth**

- “telemedicine” [MeSH Terms] OR “telemedicine” OR “mhealth” OR “mobile health units” [MeSH Terms] OR “mobile health units” OR “cell phone” [MeSH Terms] OR “cell phone” OR “smartphone” [MeSH Terms] OR “smartphone” OR “satellite phone” OR “computers, handheld” [MeSH Terms] OR “computers, handheld” OR “tablet computer” OR “mobile application” OR “mobile app” OR ”apps” OR “eHealth” OR “computers” [MeSH Terms] OR “computers” [All Fields] OR “internet-based intervention” [MeSH Terms] OR “internet-based intervention” OR “internet” OR “internet-based” OR “web-based” OR “text messaging” [MeSH Terms] OR “text messaging” OR “telehealth.”

### Eligibility Criteria

We included all interventional studies published from inception to November 3, 2022, limited to peer-reviewed articles that were written in English and conducted among humans. We considered the inclusion of literature in this review if it met the following criteria: (1) it dealt with all adults (18 years and older) diagnosed with NAFLD using sonography or transient elastography (FibroScan, Echosens); (2) any eHealth interventions for lifestyle modification related to diet or physical activity, delivered through an electronic device with internet connectivity or wireless capacity, were used; and (3) outcomes were reported as any quantifiable measure for evaluating the effect of lifestyle modification (body weight, BMI, alanine aminotransferase [ALT], or aspartate aminotransferase [AST]).

The exclusion criteria were as follows: (1) if patients with NAFLD were not the main participants, (2) if NAFLD was not the primary diagnosis, (3) if interventions were only targeted at patients with NAFLD, and (4) if only eHealth techniques were used for evaluating the outcomes.

### Study Selection

Study citations were imported and compiled into reference management software (Endnote X9.2; Clarivate Analytics) for selection. Studies were screened and selected by 3 reviewers (JC, OYK, and YJ). For the initial search, after removing duplicates, 2 researchers (JC and OYK) independently screened the titles and abstracts of the studies identified from 5 electronic databases to remove irrelevant studies, using eligibility assessment lists. In the second phase, the researchers checked the study types of all the remaining studies, and the full texts of potentially relevant articles were retrieved. Any disagreements or discrepancies between the 2 authors (JC and OYK) regarding the inclusion of studies were resolved through discussion with a third reviewer (YJ).

### Data Extraction

Two researchers (JC and OYK) extracted and cross-checked the data independently. The following information was extracted from each selected study: author, year of publication, study location, intervention and control groups (age, sex, and sample size), details about intervention and control (device, intervention and control type, intervention content, and follow-up duration), and relevant outcomes and results. If the studies reported that data were insufficient for meta-analysis, the lacking data were requested from the corresponding authors. All the researchers checked the extracted data for consistency. Discrepancies were resolved through discussions.

### Data Synthesis and Analysis

Descriptive analyses of the data were performed, and the findings were synthesized based on the category of outcomes, intervention type and content, and its effect. The primary outcomes of this review were weight, BMI, and liver enzyme levels. When an original study reported only the total sample size of the follow-up data without the number of each control and intervention group, we estimated the sample size of each group using pooled standard deviation formula with pooled SD of each group and 95% CI [22].

Meta-analysis of the extracted data was conducted using the Review Manager of the Cochrane Collaboration (RevMan 5.3, Cochrane Organization). Standardized mean differences (SMDs) and corresponding 95% CIs were computed as between-group differences in means divided by pooled SDs with Hedges correction [23]. The Cochrane Handbook states that the pooled SMDs are equal to the effect size.

Heterogeneity within studies was calculated using the *I*^2^ heterogeneity degree, wherein 20%, 50%, and 75% were considered indicative of low, medium, and high heterogeneity, respectively [23]. We used a fixed effect model for meta-analyses. If heterogeneity, as measured by *I*^2^ heterogeneity degree, was greater than 50% and *P*<.1, we performed random-effects analysis.

### Risk of Bias Assessment

The quality of included studies was assessed by using the Cochrane Collaboration Risk of Bias Tool (RoB II) for included randomized controlled trials (RCT) [23]. For nonrandomized controlled studies, the risk of bias assessment tool for nonrandomized studies developed by the Korean National Evidence-Based Healthcare Collaborating Agency was used to evaluate the risk of bias [24]. Two researchers (JY and OYK) independently completed the bias assessment, and any disagreement was resolved through consultation with a third researcher (YJ).

For RCTs, the following criteria were considered: (1) the randomization process, (2) deviations from intended interventions, (3) missing outcome data, (4) measurement of the outcome, and (5) selection of the reported result. Each domain was evaluated using the Cochrane risk ratings of “low,” “high,” and “some concerns.” The criteria considered for the quasi-experimental trial included (1) the selection of participants, (2) confounding variables, (3) the measurement of exposure, (4) blinding of the outcome assessments, (5) incomplete outcome data, and (6) selective outcome reporting. Risk ratings of “low,” “high,” and “unclear” were assigned for each bias.

## Results

### Study Selection

**Figure 1:**
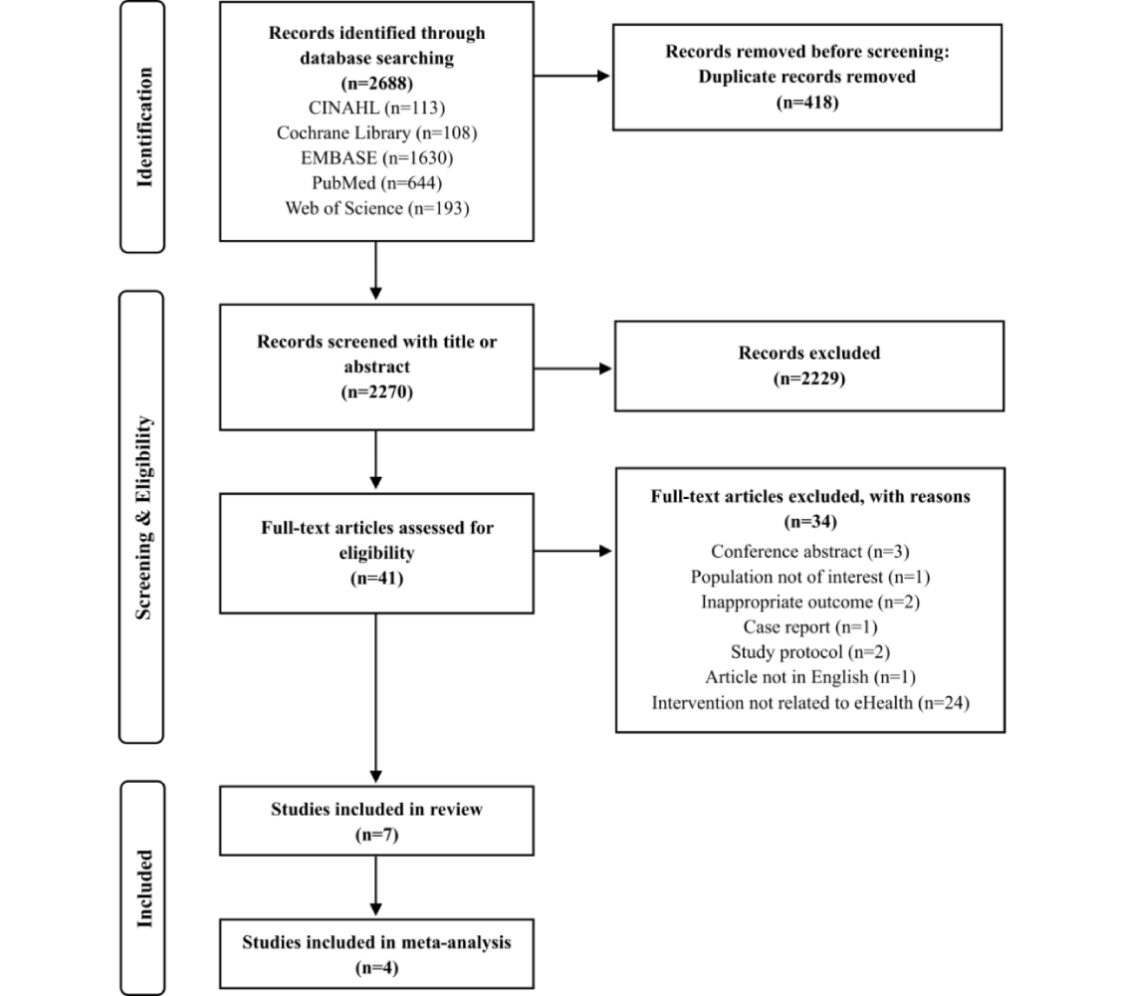
PRISMA (Preferred Reporting Items for Systematic Reviews and Meta-Analyses) flow diagram.

Our initial search identified 2688 records from 5 relevant database searches (Figure 1). After excluding duplicates, 2270 articles were screened by title and abstract, resulting in the exclusion of a further 2229. We assessed the full text of 41 potentially eligible papers, and 8 of those met the inclusion criteria. Of those, the studies by Pfirrmann et al [25] and Huber et al [26] used the same intervention program and study population, which was taken into account. Seven intervention studies [25,27-32] were consequently included in this review. All included studies were clinical trials: 4 were RCTs [27-30] and 3 were non-RCTs [25,31,32]. All 7 studies were included in the descriptive synthesis. From among these, 4 studies that were conducted using random allocation were included in the meta-analysis.

### Characteristics of Included Studies

The characteristics of the included studies are summarized in Table 1. The studies were published during 2016-2022 in 6 geographical regions (China, Germany, Iran, Italy, Singapore, and the United States).

This review encompassed 1257 participants [25,27-32]; 498 (39.6%) were recruited from gastrointestinal clinics affiliated to a hospital [27-30,32] and 759 (60.4%) were enlisted from either the center of Metabolic Diseases and Clinical Dietetics or Sports Medicine, which serves as the second-level center [25,31]. The average age of the participants in the studies ranged from 38.3 to 57.9 years, and 65.1% (818/1257) were male. One study recruited only male participants [28]. The duration of the intervention ranged from 3 to 24 months. The mean sample size was 180 individuals, the lowest number being 30, and the highest 716.

The majority of participants had NAFLD, regardless of whether they were overweight, obese, or diabetic. However, Lim et al [30] only recruited patients diagnosed with NAFLD who had a BMI ≥ 23 kg/m^2^.

**Table 1:** Characteristics of the included studies.

Study	Country	Study design	Study participants, sample size (n)	Participants age (years), mean (SD)	Duration (months)
Dong et al [28], 2016	China	RCT^a^	260 patients with NAFLD^b^ IG^c^: n=130 CG^d^: n=130	IG: 57.9 (5.3) CG: 56.7 (5.7)	24
Fard et al [29], 2016	Iran	RCT	60 patients with NAFLD IG: n=30 CG: n=30	IG: 40.3 CG: 38.3	3
Axley et al [27], 2017	United States	RCT	30 patients with NAFLD IG: n=17 CG: n=13	IG: 54 (2.7) CG: 52 (2.3)	6
Lim et al [30], 2020	Singapore	RCT	108 patients with NAFLD IG: n=55 CG: n=53	IG: 46.8 (11.1) CG: 46.2 (10.1)	6
Mazzotti et al [31], 2018	Italy	Non-RCT	716 patients with NAFLD IG: n=278 CG: n=438	IG: 46 (11.5) CG: 55.1 (12.3)	3
Pfirrmann et al [25], 2019	Germany	Non-RCT	43 patients with NAFLD IG: n=43	IG: 43 (10.9)	3
Tincopa et al [32], 2022	United States	Non-RCT	40 patients with NAFLD IG: n=40	IG: 52.5	6

^a^RCT: randomized controlled trial.

^b^NAFLD: nonalcoholic fatty liver disease.

^c^IG: intervention group.

^d^CG: control group.

### Intervention Characteristics

The characteristics of the interventions are presented in Table 2. Of the included 7 studies, 3 studies [28,29,31] provided professional counseling of diet and physical activity to both intervention and control group. The intervention group additionally administrated a contact-free visit or web-based contact by health professionals for providing health guidance or following up on recommended information. Two interventions [27,30] operated additional intervention components only to the intervention group, while providing standard care for liver disease in the clinic to the control group. Two studies mainly provided intervention on exercise to the intervention group [25], and one of the studies included nutritional assessment [32].

All studies measured changes in body weight, BMI, and liver enzymes [25,27-32]. Five of the studies used weight loss as the main outcome [25,27,28,31,32], and 1 study reported liver enzymes as the primary outcome [29] and 1, a pilot study, assessed drop and completion rates [32]. Six studies measured the body weight of participants by trained personnel using standardized procedures and equipment, but 1 study did not report the measurement methods [32]. Five of the included studies reported that the effect was statistically significant in the intervention group compared to the control group. One study that used a single-arm design showed significant differences in body weight and BMI compared to baseline [25].

**Table 2:** Characteristics of interventions and their effect.

Study	Description of the study group		Intervention effect
	Intervention group	Control group	
Dong et al [28], 2016	Lifestyle counseling for diet and physical activity by 2 professional physicians Phone visit by doctors (10 min) every 3 months, providing health guidance on diet and exercise	Lifestyle counseling for diet and physical activity by 2 professional physicians	Weight: +^a^ (IG^b^), BMI: + (IG, CG^c^), AC^d^: + (IG), ALT^e^: + (IG), AST^f^: −^g^, NAFLD-FS^h^: + (IG, CG), liver steatosis grade: + (IG, CG)
Fard et al [29], 2016	Counseling, dietary advice from a nutritionist performing physical activities, face-to-face consultation Telephone call follow-up the recommended diet and physical activities	Counseling, dietary advice from a nutritionist performing physical activities, face-to-face consultation	Weight: + (IG), BMI: + (IG), ALT: + (IG), AST: + (IG)
Axley et al [27], 2017	SMS text messages provided information and education including individual goal, nutrition, exercise, stress management, behavior change, and overcoming barriers every 9 AM	Standard of care for liver disease with detailed instruction in clinic on a healthy diet and daily exercise for weight loss	Weight: + (IG), BMI: + (IG), ALT: + (IG), AST: + (IG), HDL^i^: −, TG^j^: + (IG)
Lim et al [30], 2020	Advice on dietary and physical activity modification by a dietician Food diary Self-monitoring of physical activity Real-time feedback and encouragement by a dietitian Peer support chat channel Weekly educational clips	Usual standard care: a single face-to-face session in the clinic	Weight: + (IG), BMI: + (IG), Waist circumference: + (IG, CG), ALT: + (IG), AST: + (IG)
Mazzotti et al [31], 2018	Web-based intervention: group program similar to GBI, divided 4 sessions using a Cloud/SaaS e-learning platform: role-game measuring adherence, motivation to change, and competence by online questionnaires; channel to interact with the clinical center offline	Group-based intervention: group counseling on diet, and habitual physical activity physicians and dietitians	Weight: + (IG), 10% weight loss: −, Waist circumference: + (IG), GGT^k^: + (IG), ALT: + (IG)
Pfirrmann et al [25], 2019	Web-based platform: Individualized training support (moderate-intensity exercise of 3 sessions per week for 4 weeks, and intensified exercise of 5 sessions per week for the remaining 4 weeks) Regular individual patient feedback Frequent interaction with a counselor and peer support using a discussion board and a chatroom Biweekly group training at the sports center	None	Weight: +, body fat: +
Tincopa et al [32]	Mobile technology-based lifestyle intervention: step count and personalized feedback on physical activity with tailored weekly step count goals using Fitbit, and motivational messages via email Nutrition assessment at baseline and month 3 by phone Overall feedback on physical activity and nutrition over the first 3 months with nutrition assessment at month 3 using phone	None	Weight: −, BMI: −, Liver fat: −, Waist circumference: +, HDL: +, LDL: +, TG: +, HbA_1c_^l^: +

^a^+: yes.

^b^IG: intervention group.

^c^CG: control group.

^d^AC: abdominal circumference.

^e^ALT: alanine aminotransferase.

^f^AST: aspartate aminotransferase.

^g^–: no.

^h^NAFLD-FS: nonalcoholic fatty liver disease fibrosis score.

^i^HDL: high-density lipoprotein.

^j^TG: triglyceride.

^k^GGT: gamma-glutamyl transferase.

^l^HbA_1c_: hemoglobin A_1c_.

### Intervention Delivery and Frequency

This review classified the intervention features of lifestyle modification of NAFLD into 8 components: initial assessment, information on NAFLD, education related to disease or healthy lifestyle, goal setting, counseling of diet and physical activity, reminders for self-management behaviors, feedback, and interactions as social support (Table 3). Each intervention used at least 4 components. All studies conducted initial assessments of participants’ physical condition, laboratory data, or self-management [25,27-32], and 6 studies set individual goals [25,27,28,30-32]. Six studies provided education on a healthy diet and physical activity for weight loss [25,27,29-32]. One study provided biweekly group exercise training at a sports center [25]. Counseling with health guidance or feedback on diet and exercise logs was conducted between participants and health professionals in 5 of the studies, using phone calls [28,29], web discussion boards [25], regular messages [30], and emails [31] for goal setting, behavior change, and encouragement. Two studies issued reminders to log meal intakes every day and weight twice a week [25,30]. Three studies provided support systems to bolster user motivation using chat channels and to improve social support and adherence by using a discussion board [25,30,31]. Two interventions were made by telephone to follow-up on guidance of the healthy diets and physical activities for NAFLD self-management [28,29], and 1 intervention used SMS text messages that generated automated or customized messages to provide education and to encourage participants [27]. Only one intervention used interactive mobile phone apps [30]. Two studies were conducted using web-based platforms that could accommodate group sessions and also provide individual counsel [25,31].

The intervention frequency varied according to the contents of each study. Most messages for facilitating lifestyle behaviors or motivational information were sent daily [27,30], and education on diet and exercise was provided 1-7 times per week. Feedback about the adherence or performance of participants was provided with various frequencies using a web platform, phone, or mobile phone.

**Table 3:** Component of the interventions.

Study	Delivery mode	Component of intervention									Frequency
		A^a^	I^b^	E^c^	GS^d^	C^e^	R^f^	F^g^	IN^h^		
Dong et al [28], 2016	Telephone	✓	✓	—^i^	✓	✓	—	✓	—	Every 3 months	
Fard et al [29], 2016	Telephone	✓	✓	✓	—	✓	—	✓	—	1-3 times per week	
Axley et al [27], 2017	SMS text message	✓	✓	✓	✓	—	—	✓	—	Daily	
Lim et al [30], 2020	Mobile app	✓	✓	✓	✓	✓	✓	✓	✓	Daily	
Mazzotti et al [31], 2018	Web-based platform	✓	✓	✓	✓	✓	—	—	✓	Weekly	
Pfirrmann et al [25], 2019	Web-based platform	✓	✓	✓	✓	✓	✓	✓	✓	5 sessions per week	
Tincopa et al [32]	Mobile technology-based	✓	—	✓	✓	—	—	✓	—	Weekly	

^a^A: assessment.

^b^I: information.

^c^E: education.

^d^GS: goal setting.

^e^C: counseling.

^f^R: reminder.

^g^F: feedback.

^h^IN: interaction.

^i^None.

### Meta-analysis of Weight, BMI, ALT, and AST

Out of 7 studies, 4 were included in the meta-analysis [27-30]. Regarding the 3 studies that have been excluded, 1 study did not apply a random allocation [31] and 2 were conducted with a single arm [25,32]. Four studies reported body weight, BMI, ALT, and AST as outcomes. However, 1 study did not report the SD of body weight and BMI. We tried to contact the authors for updating the insufficient data but did not get any response and excluded the study of the relevant analyses [29]. As a result, 3 interventions were shown in the forest plot of body weight and BMI [27,28,30], and 4 interventions for ALT and AST [27-30].

**Figure 2:**
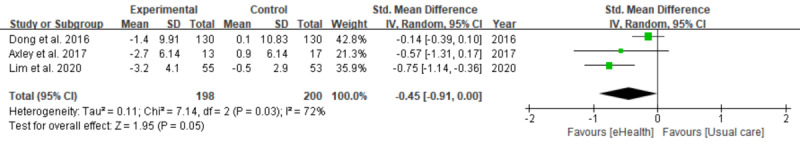
Forest plot of the difference of body weight between the intervention and the control group.

**Figure 3:**
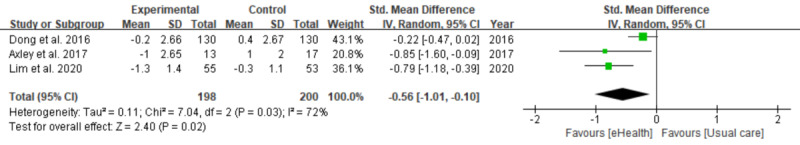
Forest plot of the difference in BMI between the intervention and the control group.

**Figure 4:**
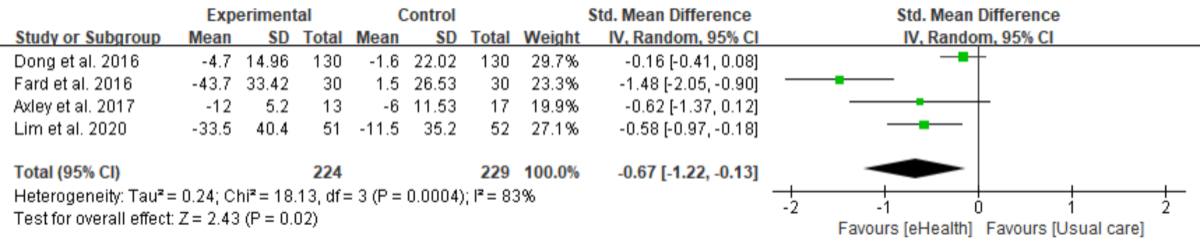
Forest plot of the difference in alanine aminotransferase (ALT) between the intervention and the control group.

**Figure 5:**
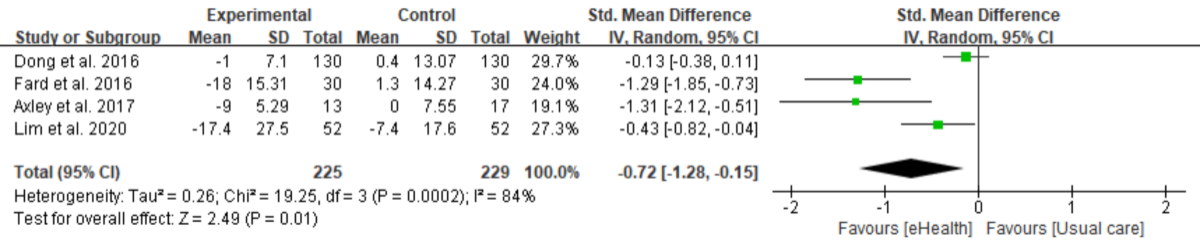
Forest plot of the difference in aspartate aminotransferase (AST) between the intervention and the control group.

As shown in Figure 2, the estimated SMD of body weight between the intervention and control groups was not significant as 0.45 (95% CI 0.91 to 0.01; *P*=.05), with a moderate level of heterogeneity (*I*^2^=72%). There was a statistically significant difference in BMI (SMD −0.56, 95% CI 1.01 to 0.10; *P*=.02) between the intervention and control groups, as shown in the forest plot, with a moderate level of heterogeneity (*I*^2^=72%; Figure 3). Significant differences were also found between the 3 groups measuring the effects of lifestyle modification interventions on ALT (SMD 0.67, 95% CI 1.22 to 0.13; *P*=.02; Figure 4) and AST (SMD −0.72, 95% CI −1.28 to −0.15; *P*=.01; Figure 5). There was a high level of heterogeneity between the studies (*I*^2^=83%; *I*^2^=84%). Multimedia Appendix 2 provides all results of the meta-analysis.

### Risk of Bias

**Figure 6:**
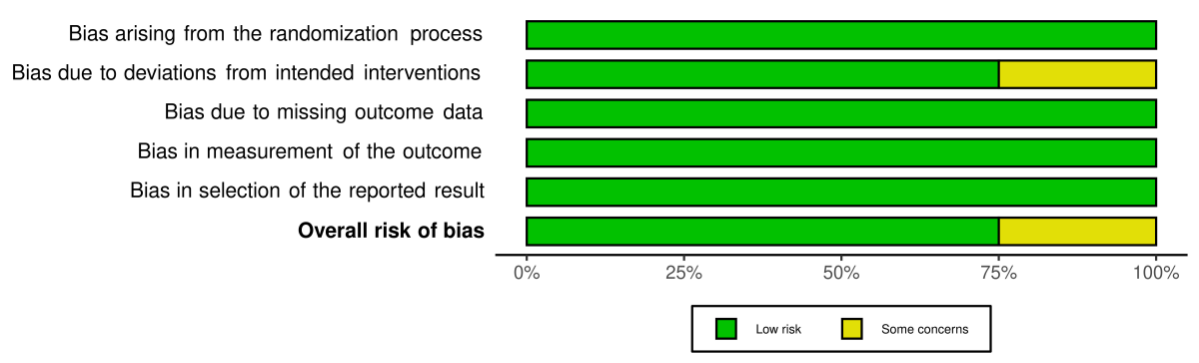
Summary of the risk of bias for randomized controlled trials.

**Figure 7:**
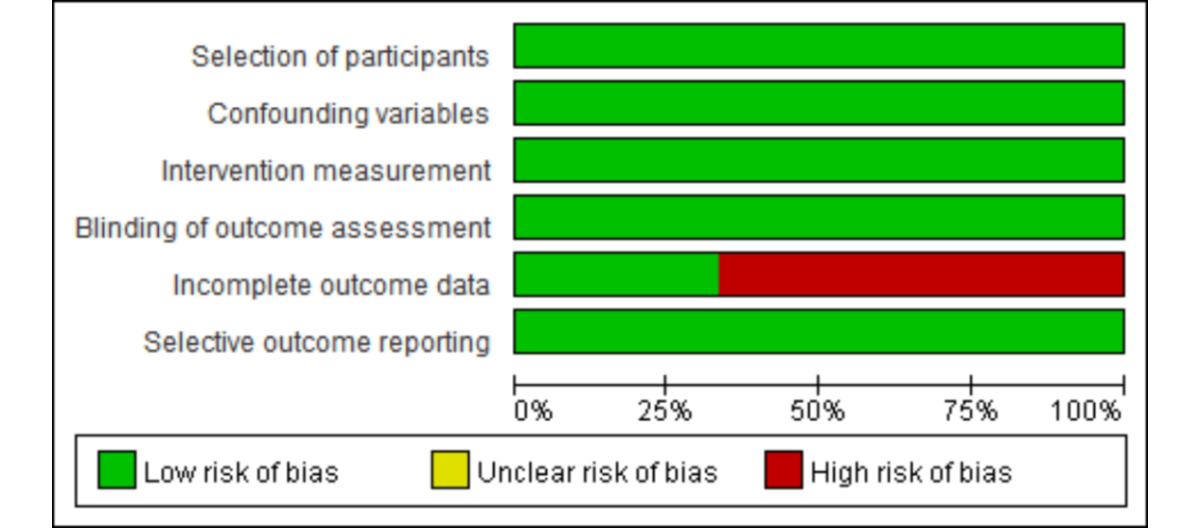
Summary of the risk of bias for nonrandomized controlled trials.

The details of the 4 RCTs’ risk of bias are summarized in Figure 6 [27-30]. Overall, the majority of studies were classified as having a low risk of bias across all main sources of bias and adopted a randomization process, measurement, and reporting of the outcome. Among all studies, 1 RCT identified “some concerns” because of the assessment of the probability of deviations from intended interventions [29]. The risk of bias assessments for the 3 non-RCTs is presented in Figure 7 [25,31,32]. All interventions were found to be “Low risk,” except for “Incomplete outcome data,” for which bias was found in 2 studies [31,32].

## Discussion

### Principal Findings

This systematic review summarized the results for the effectiveness of eHealth interventions for lifestyle modifications in body weight, BMI, ALT, and AST among adult patients with NAFLD. All the interventions were published between 2016 and 2022. This shows that eHealth technology is not yet widely applied in NAFLD treatment interventions. This study is therefore significant, as it is the first review to identify the effect of eHealth-enabled interventions for supporting sustainable NAFLD management.

Interventions using eHealth for NAFLD were beneficial at achieving weight loss and effective in improving BMI according to the findings of our systematic review and meta-analysis. The NAFLD guideline states that weight loss is the primary goal in the treatment of NAFLD, which leads to reduced liver fat [2]. Although the eHealth interventions were not statistically effective for weight loss, the result showed a trend, which could be helpful for weight loss. The small differences in weight reduction or BMI between intervention and control groups may occur as a placebo effect under natural circumstances [33]. The results of this review are consistent with the recommendations in the guidelines and are similar to those of previous studies that verified the effect of eHealth interventions on weight loss or BMI in other populations [34,35].

The eHealth interventions to improve BMI were effective when conducted for 3 months or longer and ranged in duration from 3 to 24 months. However, in a meta-analysis of BMI, 2 of the 3 studies that were performed for 6 months were more effective than those that were performed for 24 months. Additionally, the frequency of contact using the intervention component delivered to participants varied from daily [27,30] to 1-3 times a week [29], and every 3 months [28]. The meta-analysis shows that the effect of interventions on BMI was better when delivered daily than was the case with longer intervals. Although the optimal contact frequency of participants with health professionals in the achievement of each intervention is unclear, frequent contact using eHealth may help achieve weight loss among this population. However, several frequent contacts will displease patients and require more resources in terms of time and manpower. Therefore, further reviews of optimal and cost-effective contact frequencies in eHealth should be performed to improve health outcomes in this population.

Lifestyle modification approaches use various intervention contents, such as assessment of participants’ lifestyle behaviors, information, education, goal setting, counseling, and feedback. In particular, most of the interventions included in the meta-analysis to examine the effectiveness of outcomes consisted of the assessment of health conditions, information on disease treatment or healthy behavior, goal setting of the behavior, and feedback on performance or self-monitoring. The effectiveness of these strategies in promoting changes in unhealthy lifestyle behaviors has been presented in previous eHealth interventions on weight management in other diseases [36,37]. It is well known that NAFLD has no disease-specific symptoms unless it progresses to severe liver diseases such as liver cirrhosis. Therefore, patients with NAFLD may lack motivation for or adherence to disease management. Interventions using eHealth with various contents would motivate their behavior and ultimately enhance sustainable NAFLD management.

Furthermore, the results of this review show that lifestyle modification interventions enabled eHealth to significantly improve biomarkers related to liver health. Some studies evaluating improvements in liver histology resulting from weight loss through lifestyle modification have reported significantly reduced ALT and AST [38-42], and identified combinations of the effective level of diet and exercise to achieve the outcome. However, these results were not consistent in all of the related studies [43-45], and it is known that generally, the levels of liver enzymes are not elevated in patients with simple steatosis [46]. Although the significance levels of liver enzymes were moderate or above in this meta-analysis, further studies are needed to analyze the biomarkers that clinically indicate the improvement of NAFLD.

Lifestyle modification, in the absence of pharmacological treatment, is important for patients with NAFLD to treat their health condition; it should be continued with a multidisciplinary team approach including health care professionals, dietitians, and exercise experts to motivate and guide these patients. This study highlights that eHealth technology-based lifestyle modifications may help not only with weight loss but also in improving unhealthy liver conditions through the control of liver enzymes.

### Strengths and Limitations of the Study

This study included clinical trials of lifestyle modification programs delivered by eHealth for the self-management of NAFLD and the first systematic review that analyzed the characteristics and effects using meta-analysis. Additionally, the results add to the existing knowledge on the improvement of NAFLD management.

This systematic review and meta-analysis have some limitations. First, the number of analyzed studies was small. This may reduce the generalizability of the findings and make it difficult to recommend the use of eHealth in clinical trials. Second, self-reporting of compliance related to diet and exercise potentially has a probability of recall and social desirability bias. This bias would affect the effectiveness of the outcomes. Lastly, the duration of the included studies ranges from 3 to 24 months. Thus, more evidence is needed to confirm the duration to improve outcomes of eHealth interventions in this population.

### Conclusion

This systematic review and meta-analysis aimed to identify and evaluate the effectiveness of eHealth interventions on lifestyle modifications in patients with NAFLD. The results demonstrated that an eHealth intervention was effective in improving body weight, BMI, and ALT and AST levels among these participants. In conclusion, eHealth technology has proven to be a potentially useful intervention strategy for modification of diet and exercise habits and will be an important facilitator to improve self-management skills for patients with NAFLD.

## Appendix

Multimedia Appendix 1 Search strategy.

Multimedia Appendix 2 Meta-analysis, forest plot, and quality assessment.

## Abbreviations

ALT: alanine aminotransferase
AST: aspartate aminotransferase
MeSH: Medical Subject Headings
NAFLD: nonalcoholic fatty liver disease
RCT: randomized controlled trial
