# Supplementary material for: The Effectiveness of eHealth Interventions on Lifestyle Modification in Patients With Nonalcoholic Fatty Liver Disease: Systematic Review and Meta-analysis
Source: J Med Internet Res. 2023 Jan 23;25:e37487. doi: 10.2196/37487 (PMC9903182; doi:10.2196/37487)
Supplement: Multimedia Appendix 1 [file jmir_v25i1e37487_app1.docx]

**Multimedia Appendix 1. Search term.**
