# Supplementary figures and images for: The Effectiveness of eHealth Interventions on Lifestyle Modification in Patients With Nonalcoholic Fatty Liver Disease: Systematic Review and Meta-analysis
Source: J Med Internet Res. 2023 Jan 23;25:e37487. doi: 10.2196/37487 (PMC9903182; doi:10.2196/37487)

**Mutlimedia appendix 2: Meta-analysis and forest plot**

Figure 2.


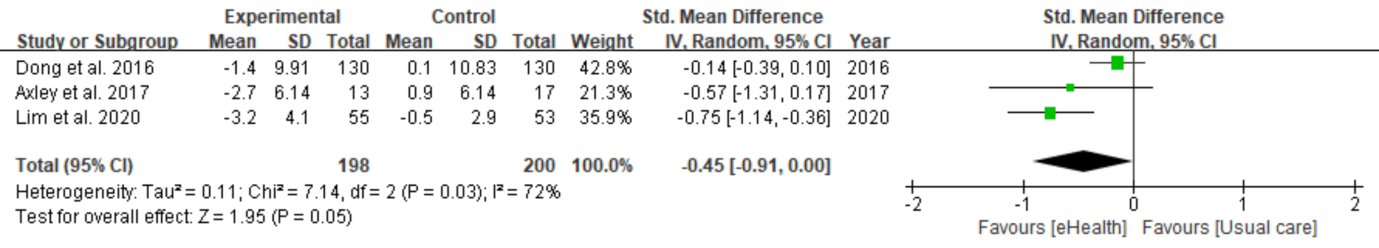


Figure 3.


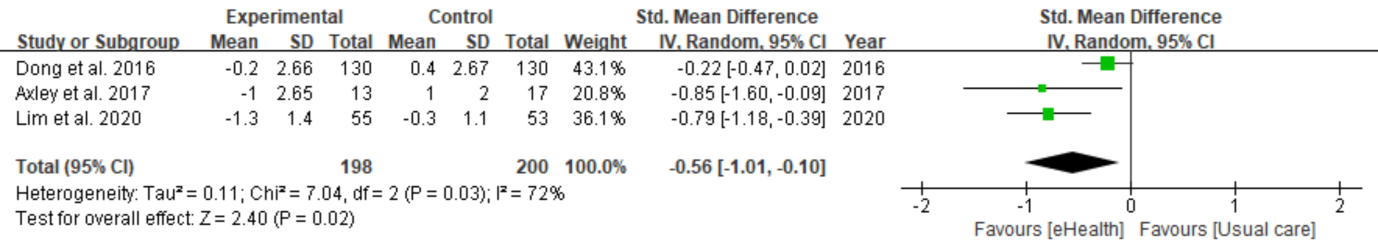


Figure 4.


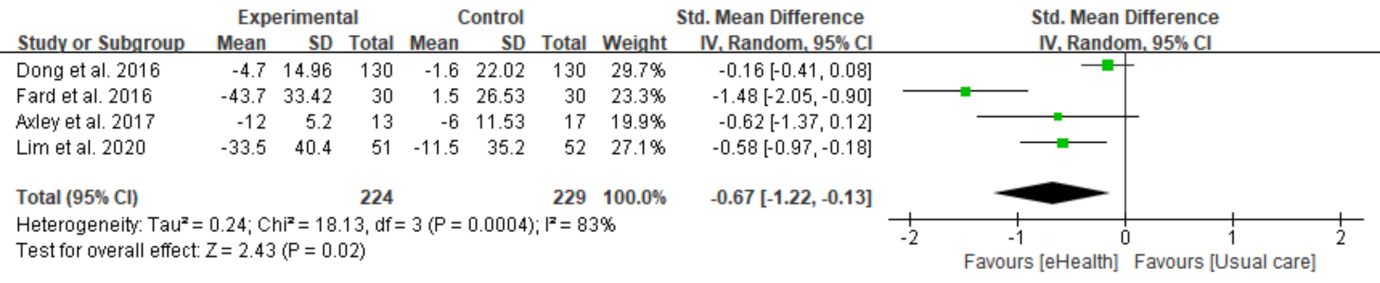


Figure 5.


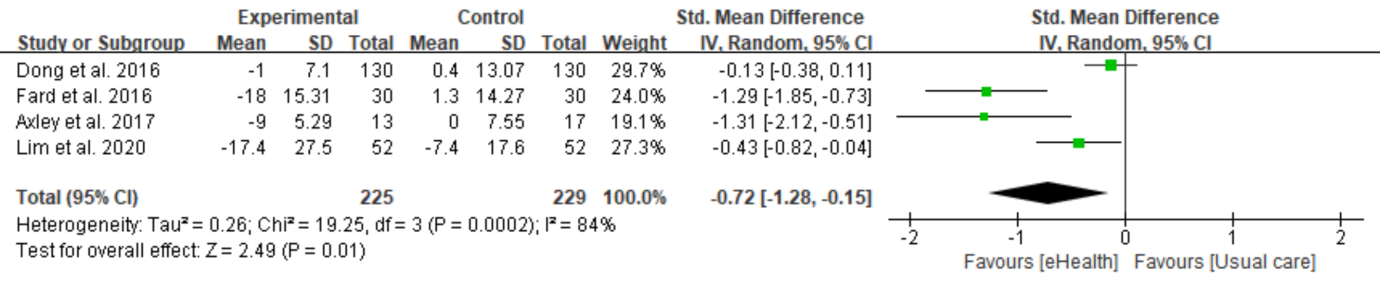


Figure 6.


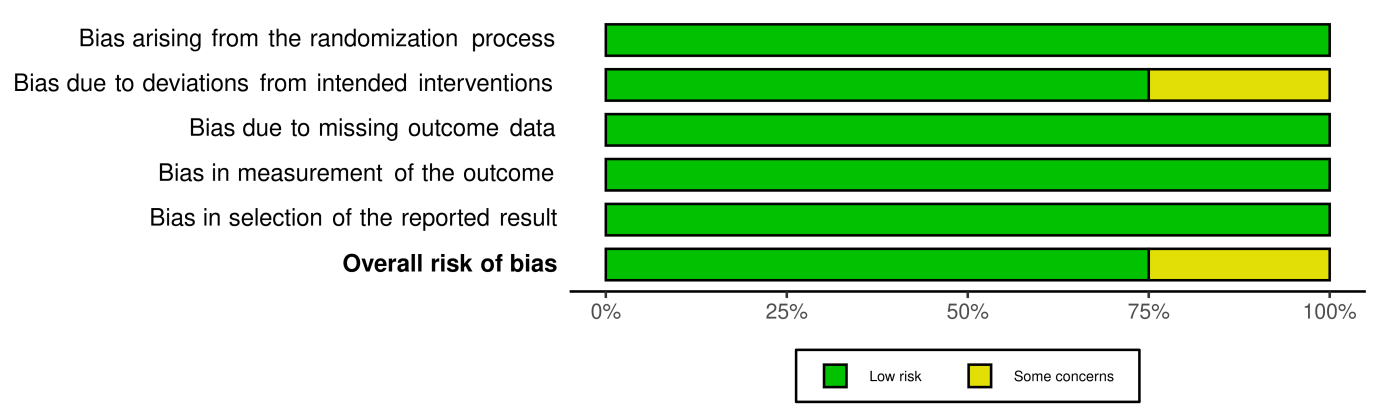


Figure 7.


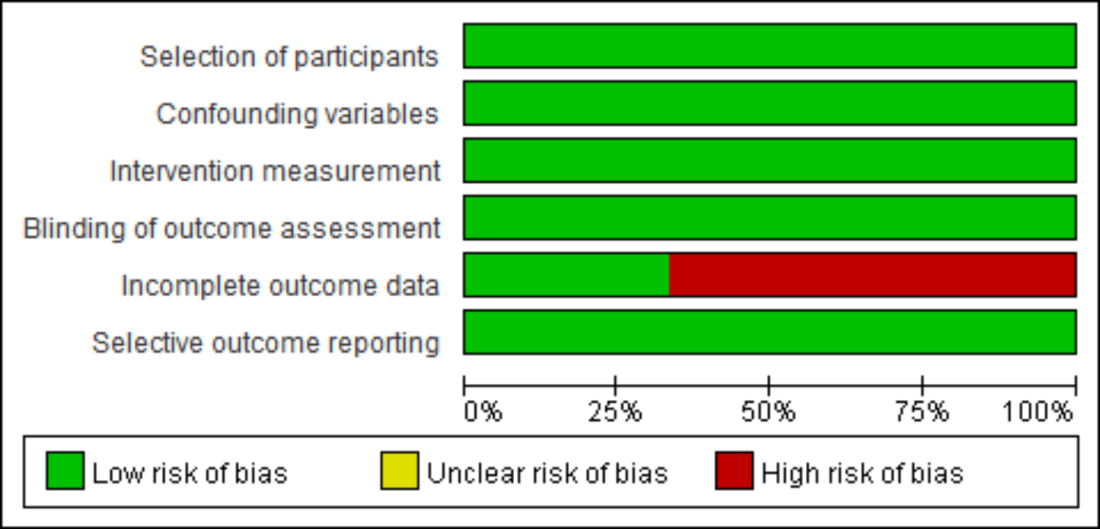

Supplement: Multimedia Appendix 2 [file jmir_v25i1e37487_app2.docx]
